# Analysis of the influence of pyroptosis-related genes on molecular characteristics in patients with acute myocardial infarction

**DOI:** 10.1097/MD.0000000000033620

**Published:** 2023-04-21

**Authors:** Huan Wu, Xiaoman Xiong, Xueying CUI, Jianlong Xiong, Yan Zhang, Liubo Xiang, TAO Xu

**Affiliations:** a School of Preclinical Medicine, Guizhou University of Traditional Chinese Medicine, Guiyang, Guizhou, China; b Qingyun County People’s Hospital, Qingyun, Shandong, China.

**Keywords:** acute myocardial infarction, molecular characteristics, pyroptosis, Random Forest, Support Vector Machine

## Abstract

Pyroptosis is a newly identified mode of programmed cell death, but the potential role in patients with acute myocardial infarction (AMI) remains unclear. In this study, bioinformatics methods were used to identify differentially expressed genes from peripheral blood transcriptome data between normal subjects and patients with AMI which were downloaded by the Gene Expression Omnibus database. Comparing Random Forest (RF) and Support Vector Machine (SVM) training algorithms were used to identify pyroptosis-related genes, predicting patients with AMI by nomogram based on informative genes. Moreover, clustering was used to amplify the feature of pyroptosis, in order to facilitate analysis distinct biological differences. Diversity analysis indicated that a majority of pyroptosis-related genes are expressed at higher levels in patients with AMI. The receiver operating characteristic curves show that the RF model is more responsive than the SVM machine learning model to the pyroptosis characteristics of these patients in vivo. We obtained a column line graph diagnostic model which was developed based on 19 genes established by the RF model. After the consensus clustering algorithm of single sample Gene Set Enrichment Analysis and Kyoto Encyclopedia of Genes and Genomes (KEGG) Enrichment Analysis, the results for them found that pyroptosis-related genes mediate the activation of multiple immune cells and many inflammatory pathways in the body. We used RF and SVM algorithms to determine 19 pyroptosis-related genes and evaluate their immunological effects in patients with AMI. We also constructed a series of by nomogram related to pyroptosis-related genes to predict the risk of developing AMI.

## 1. Introduction

Acute myocardial infarction (AMI), the most dangerous type of coronary heart disease, is currently one of the leading causes of human death worldwide.^[1]^ With the development and availability of treatments such as intravenous thrombolysis and percutaneous coronary intervention, the death rate of AMI has decreased significantly, but the risk of major adverse cardiovascular events including heart failure after AMI has increased evidently.^[2]^ The search for new biomarkers and prognostic genes has therefore become a research trend in the age of precision medicine.

Pyroptosis is a newly discovered form of programmed cell death that is activated in the early stages of AMI and further amplifies the pro-inflammatory response in the body.^[3]^ Pyroptosis is characterized by cell membrane pore formation, cytoplasmic swelling and cell membrane rupture, leading to the release of pro-inflammatory factors and cellular contents. It could be observed in macrophages, cardiomyocytes and fibroblasts in the peripheral circulatory system. This aseptic inflammatory injury plays an important role in the development of AMI, but the potential mechanism of action is not clearly visible.^[4]^

With the development and application of high-throughput sequencing technology, these accessed data such transcriptome information of many disease pathologies will considerably improve mankind exploration of the mechanisms and patterns of disease onset, development and regression.^[5,6]^ Based on high-throughput sequencing data, this study used a combination of bioinformatics and machine learning methods to screen for biomarkers and molecular mechanisms of pyroptosis genes in AMI pathology, with a view to providing new ideas for the prevention and treatment of AMI.

## 2. Materials and methods

**Figure 1.**
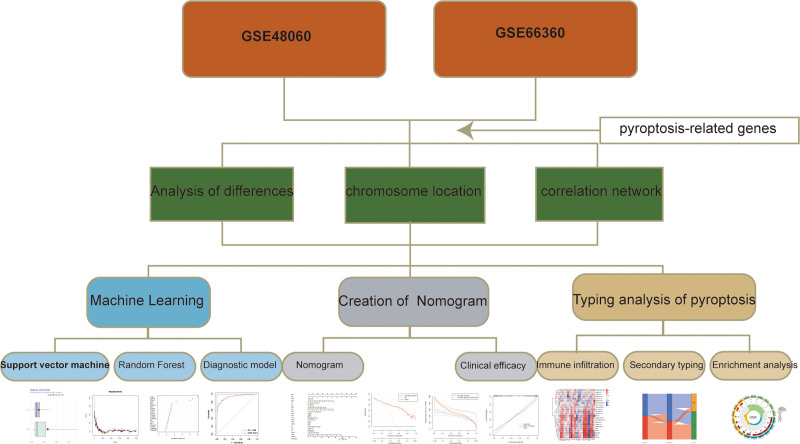
Flow chart of research design.

The overall design of the study was presented in Figure 1.

### 2.1. Collection and processing of data

Download the GSE48060, GSE66360 datasets and GPL570 [HG-U133_Plus_2] Affymetrix Human Genome U133 Plus 2.0 Array platform annotations file in the gene expression omnibus database (https://www.ncbi.nlm.nih.gov/geo/). The mRNA expression matrices were obtained by writing Perl scripts to annotate the probes of GSE48060, GSE66360 expression matrices with the platform. The 2 datasets were then merged after the removal of batch effects using the System Verilog Assertion.

### 2.2. Expression of pyroptosis-related genes in AMI patients

Expression of pyroptosis-related genes in the dataset was extracted by R language and analyzed for differences using the limma, charting the location of these genes on chromosomes based on human chromosome location. The expression of pyroptosis-related genes was analyzed to plot heatmaps and correlation networks for visualization.

### 2.3. Comparison of models for neural networks and machine learning

Construction of Random Forest (RF) models and machine learning models is carried out with the packages of RF algorithm and Kernel Methods in R language,^[7,8]^ and multiple algorithmic models are analyzed using the DALEX package. The stability of the 2 models was verified by the results of the reverse cumulative distribution of residuals, the residual boxplot and the receiver operating characteristic curve. Candidate pyroptosis-related genes were identified based on 10-fold cross-validation and genes with importance values larger than 2 were selected as disease-specific genes, the optimal mtry and ntree parameters were finally configured to 3 and 500 for subsequent RF model construction.^[9]^

### 2.4. Creation of the nomogram

Predicting the onset of AMI by a nomogram based on disease-specific genes.^[10]^ The reliability and usefulness of this nomogram was assessed using calibration curves, decision curve analysis (DCA) and clinical impact curves (CIC).^[11]^

### 2.5. Typing analysis of pyroptosis-related genes

The samples were classified according to consensus clustering using the K-means algorithm, and the typing results were used to perform another differential analysis of the pyroptosis genes and to create an expression heatmap.^[12,13]^ Principal component analysis (PCA) was used to observe the distribution of the samples and to obtain pyroptosis score for each sample.^[9,14]^ The single sample Gene Set Enrichment Analysis was used to calculate immune cell infiltration-based signature in atherosclerotic samples.^[15]^ The R heatmaply package was used to graph the correlation between expression of pyroptosis-related genes and immune cells, and differential analysis of transcriptome-wide genes was performed based on the typing information of the samples to obtain differential genes between genotypes. Gene Ontology (GO) enrichment analysis and Kyoto Encyclopedia of Genes and Genomes (KEGG) enrichment analysis was performed by R language to observe their molecular changes in AMI. Finally, the differential genes between the different genotypes were typed again to obtain differential genotypic information. The differences in pyroptosis risk score between the groups after the 2 fractional treatments were compared, and the correspondence between pyroptosis genotyped samples, differentially genotyped samples and pyroptosis score was demonstrated by drawing a Sankey diagram with the ggalluvial package.

## 3. Results

### 3.1. Differential expression and visualization of pyroptosis-related genes

**Figure 2.**
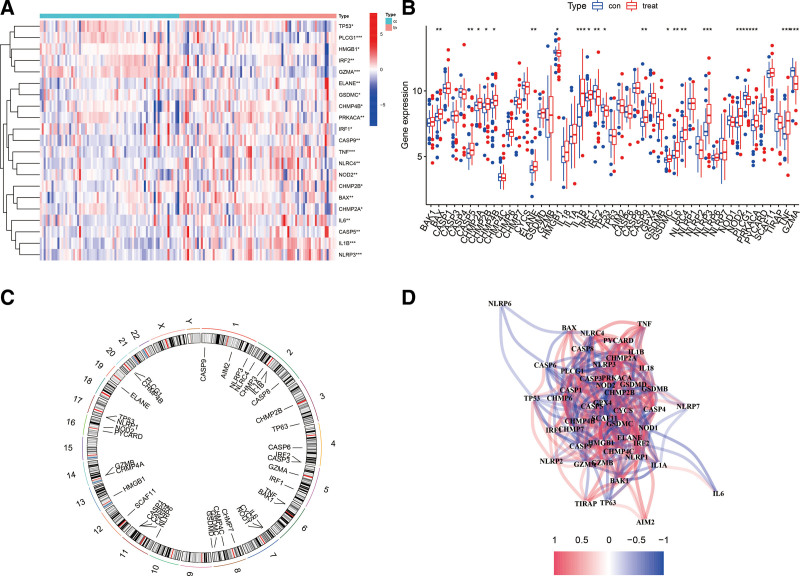
(A) Differential expression heatmap of pyroptosis-related genes in AMI patients. (B) Histogram of pyroptosis-related gene expression in the blood of normal subjects and AMI patients. (C) Distribution of pyroptosis-related genes in chromosomes. (D) Correlation analysis of the expression of pyroptosis-related genes. AMI = acute myocardial infarction.

The 2 datasets (GSE48060, GSE66360 datasets) were combined after the removal of batch effects and 52 pyroptosis-related genes were extracted. Differential analysis revealed that 21 pyroptosis genes were differentially expressed in the blood of normal subjects as compared to patients with AMI (Fig. 2A and B). The location of pyroptosis-related genes on the chromosome was plotted using the RCircos package (Fig. 2C). Correlation analysis showed a strong correlation among pyroptosis genes (Fig. 2D).

### 3.2. Comparison of RF models constructed from pyroptosis-related genes with support vector machine (SVM) models

**Figure 3.**
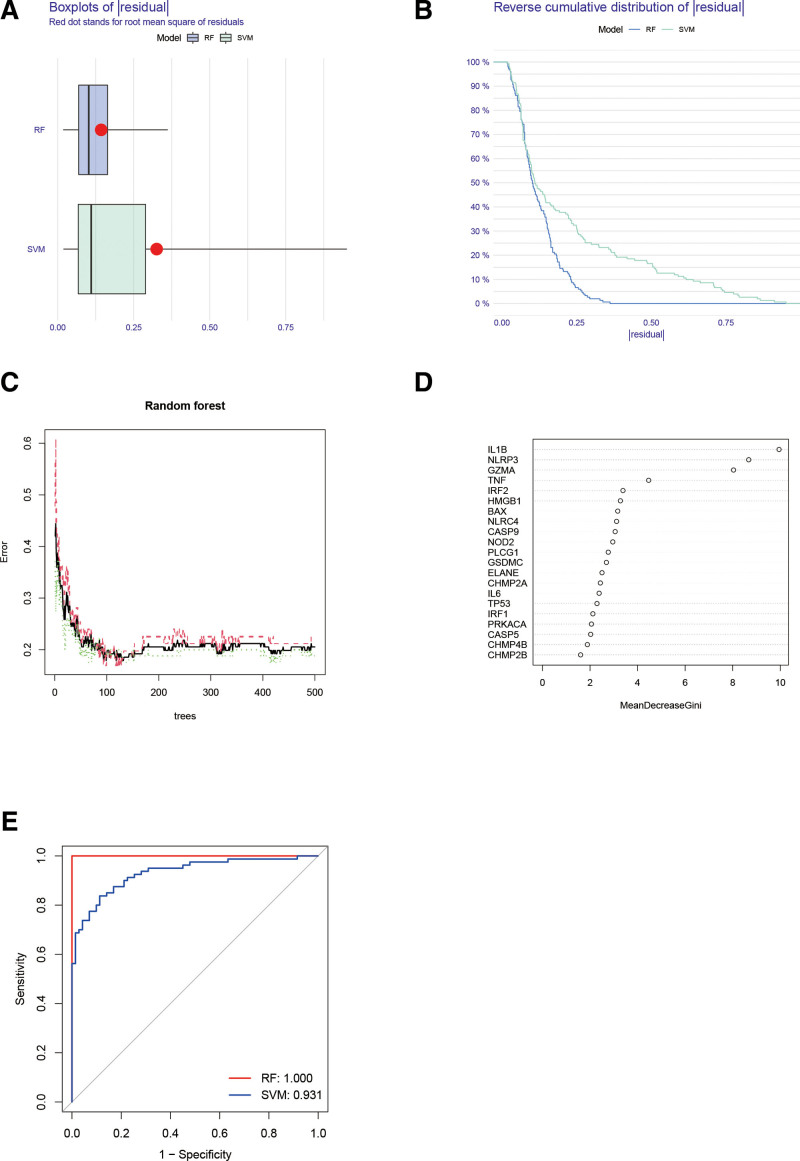
(A) Residual boxplot of RF and SVM model. (B) Reverse cumulative distribution of residuals of RF and SVM model. (C) Error plot in Random Forest. (D) Importance score for pyroptosis-related genes. (E) ROC curve of RF and SVM model. ROC = receiver operating characteristic, RF = Random Forest, SVM = Support Vector Machine.

Based on the expression of differential pyroptosis-related genes in each sample, RF and SVM machine learning algorithms were used to identify the genes with more characteristics as model genes for subsequent model construction. Both the box line plots of the residuals and the reverse cumulative distribution of the residuals were plotted to show that the error was smaller using the RF model compared to the SVM model (Fig. 3A and B).

The error plot in RF shows that both the false positive and false negative rates and the combined error rate of the RF model decrease as the number of training sessions increases (Fig. 3C). On the contrary, genes such as interleukin 1 beta (IL-1β), NOD-like receptor family pyrin domain containing 3 (NLRP3) and granzyme A (GZMA) had a greater preference in the same model (Fig. 3D). The receiver operating characteristic curves also showed that the RF model was more responsive to disease signature genes than the SVM model (Fig. 3E).

### 3.3. Nomogram construction of pyroptosis-related genes

**Figure 4.**
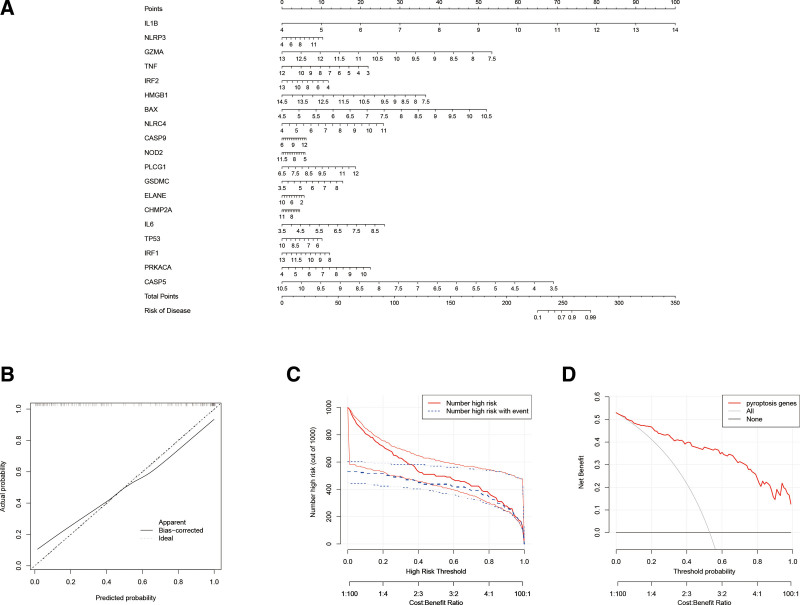
(A) Evaluation of nomogram model for pyroptosis-related genes. (B) Calibration curve of nomogram model. (C) DCA for the nomogram model. (D) Clinical impact curve based on the nomogram model. DCA = decision curve analysis.

Based on the relative importance values we selected 19 genes for the construction of the Nomogram model to predict the incidence of AMI, all of which had an importance score higher than 2. The genetic risk score was used to predict the onset of this disease (Fig. 4A). The reliability and clinical impact of the model was assessed using calibration curves, DCA and CIC, with the calibration curves being more stable (Fig. 4B).

The height of the red line of the DCA is always the highest which indicates that it is more profitable to use the Nomogram model to assess the condition of AMI patients (Fig. 4C). The CIC also show that the Nomogram model has better predictive capacity (Fig. 4D).

### 3.4. Genotyping of pyroptosis-related genes

**Figure 5.**
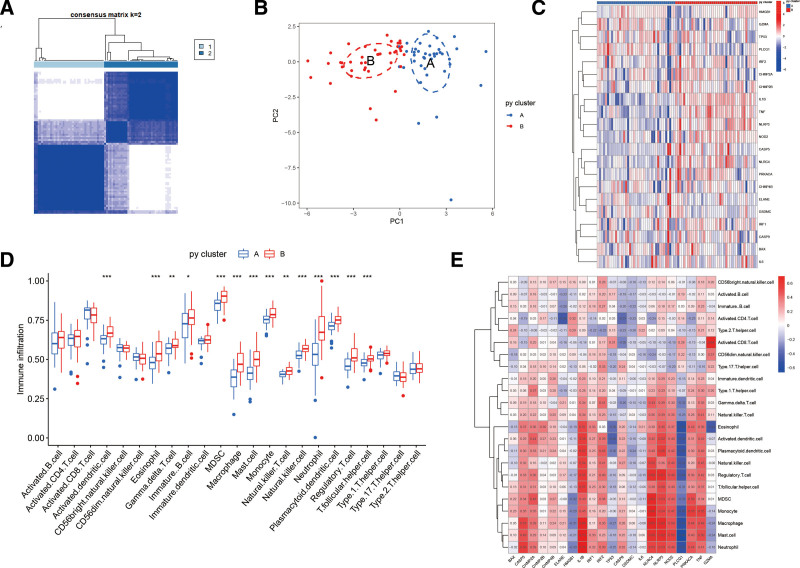
(A) Fractal matrix of pyroptosis-related genes. (B) PCA analysis of pyroptosis genotypes. (C) Differential distribution of pyroptosis-related genes in subtypes A and B. (D) Differential distribution of immune cells in subtypes A and B. (E) Heatmap of the correlation between immune genes and pyroptosis-related genes. PCA = principal component analysis.

The samples were classified into 2 types (A and B) based on the amount of pyroptosis genes expression (Fig. 5A), and the results of the PCA analysis showed a clear boundary between type A and type B (Fig. 5B). The expression of pro-inflammatory genes such as IL1β and NLRP3 was found to be higher in type B from the heatmap of pyroptosis-related genes, suggesting that type B may have a worse prognosis (Fig. 5C). Applying the single sample Gene Set Enrichment Analysis algorithm to assess the differences in immune cells between type A and type B, there were more cell subtypes such as activated dendritic cells, eosinophils, gamma delta T cells (γδ T cells), immature B cells, myeloid-derived suppressor cells (MDSC), macrophages, mast cells, monocytes, natural killer cells, neutrophils, plasmacytoid dendritic cells, regulatory T cells, T follicular helper cells, type 1 helper T cells (Th1) in type B than in type A (Fig. 5D). These differences in immune cells were closely correlated with the amount of expression of pyroptosis-related genes (Fig. 5E).

### 3.5. Establishment and evaluation of molecular subtypes

**Figure 6.**
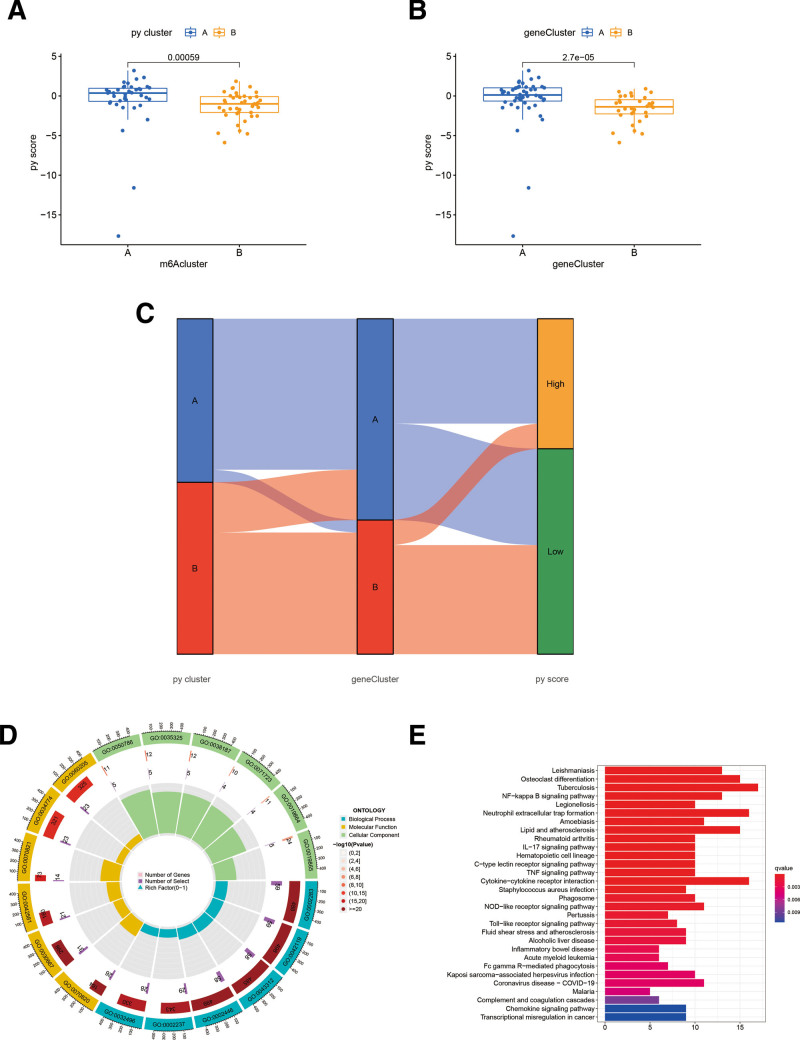
(A) Difference image in genotype scores for pyroptosis. (B) Difference image in differential genotype score. (C) Sankey diagram between differential genotype and pyroptosis genotype. (D) GO analysis of differential genes. (E) KEGG analysis of differential genes. GO = Gene Ontology, KEGG = Kyoto Encyclopedia of Genes and Genomes.

To further explore the molecular differences between pyroptosis genotypes, we analyzed the differences between the 2 sets of samples for pyroptosis genotypes. The samples were again genotyped according to differential gene expression and different samples within these 2 genotypes were examined by use of the pyroptosis score. The results indicated that both typologies captured the pyroptosis characteristics better, though the sample genetically mediated by the differential gene was superior (Fig. 6A and B). The Sankey diagram displaying the relationship between pyroptosis genotyped samples, differentially genotyped samples and pyroptosis score (Fig. 6C). GO enrichment analysis showed that differential genes are mainly participating in biological process such as neutrophil activation involved in immune response, neutrophil activation, neutrophil degranulation, neutrophil mediated immunity, response to molecule of bacterial origin, response to lipopolysaccharide, phagocytosis, cytokine secretion, leukocyte migration, leukocyte chemotaxis, positive regulation of defense response, myeloid leukocyte migration, cellular response to biotic stimulus, positive regulation of cytokine production, cell chemotaxis, in cell component such as tertiary granule, secretory granule membrane, specific granule, tertiary granule membrane, secretory granule lumen, cytoplasmic vesicle lumen, vesicle lumen, specific granule membrane, tertiary granule lumen, ficolin-1-rich granule membrane, external side of plasma membrane, ficolin-1-rich granule, ficolin-1-rich granule lumen, phagocytic vesicle, endocytic vesicle, and in regulating molecular function such as RAGE receptor binding, Toll-like receptor binding, pattern recognition receptor activity, lipopeptide binding, IgG binding, immunoglobulin binding, immune receptor activity, cytokine activity, amide binding, cytokine receptor binding, complement binding, cytokine receptor activity (Fig. 6D). KEGG enrichment analysis revealed that the differential genes were mostly enriched in NF-kappa B signaling pathway, neutrophil extracellular trap formation, lipid and atherosclerosis, IL-17 signaling pathway, hematopoietic cell lineage, C-type lectin receptor signaling pathway, TNF signaling pathway, cytokine-cytokine receptor interaction, phagosome, NOD-like receptor signaling pathway, Toll-like receptor signaling pathway (Fig. 6E).

## 4. Discussion

AMI is one of the leading causes of death worldwide. It is mostly caused by thrombosis and lumen occlusion in the coronary arteries based on coronary atherosclerosis, which results in myocardial ischemic and hypoxic injury and the development of corresponding clinical symptoms and signs.^[16]^

The process of AMI involves a complex pathology related to the deposition and denaturation of atheromatous plaques, thrombosis, ischemic and hypoxic myocardial cell injury, of which the non-bacterial inflammatory reaction mediated by pyroptosis is covering all aspects of the progression of the disease.^[17,18]^ This study identifies morbid signature genes and analyses the biological role of pyroptosis-related genes mediated in the organism by RF and SVM algorithm.

The process of pyroptosis is mostly regulated by the classical caspase-1-dependent pyroptosis and the non-caspase-1-dependent pyroptosis, with the classical caspase-1-dependent pyroptosis being more common and this pathway being dominated by the activation of NLRP3 inflammasome. Once the external stimulus is recognized by pattern recognition receptors on the cell membrane and cytoplasm, it stimulates the activation of NLRP3, absent in melanoma 2 and other pyroptosis-related proteins. It then proceeds to recruit ASC (apoptosis-associated speck-like protein containing caspase recruitment domains) and caspase-1 for splicing, forming biologically active NLRP3 inflammasome and promoting maturation and secretion of IL-1β and IL-18. Caspase-1 also cleaves gasdermin (GSDM) to release the GSDM-N structural domain, which is able to bind lipid membranes and punch holes in the cell membrane, leading to characteristic morphological changes such as cytoplasmic swelling and membrane rupture. In comparison, the non-caspase-1-dependent pyroptosis is relatively simple and is partly completed by caspase-4/5/11 activation and cleavage of GSDM following lipopolysaccharide stimulation of ASC. In addition, caspase-4/5/11 stimulates pannexin-1, which in turn induces the activation of Purinergic Receptor P2X7 on the cell membrane to form micropores. It is worth noting that activation of pannexin-1 also promotes the formation of NLRP3 inflammasome causing cell pyroptosis. It follows that classical and non-classical molecular pathways synergistically regulate the process of pyroptosis during disease progression. It is thought-provoking to realize that pyroptosis causes more severe morphological changes to the cells, resulting in a large number of bubble-like protrusions on the surface of the cell membrane, which consequently form pore membranes. The deoxyribonucleic acid, chromatin, mitochondria, lysosomes and other material inside the cell are also damaged to various degrees and released outside the cell through the pore membrane, and the cell itself tends to flatten out as the substance flows out of the cell.

We constructed a RF model based on 19 genes, including NLRP3, IL-1β, interleukin 6, high mobility group box 1 protein (HMGB1), GZMA, TNF, interferon regulatory factor 2, NLR Family CARD Domain Containing 4, Caspase 9, nucleotide binding oligomerization domain containing 2, Phospholipase C Gamma 1, Gasdermin C, elastase, neutrophil expressed, charged multivesicular body protein 2A, interferon regulatory factor 1, Protein Kinase CAMP-Activated Catalytic Subunit Alpha, Caspase 5, BCL2 associated X, apoptosis regulator (BAX), TP53. The role played by these genes in the inflammatory process has been described in some detail in the previous literature. For example, NLRP3 inflammasome are a large multi-protein complex composed of NLRP3, ASC and caspase-1 proteins. Over-activation of NLRP3 inflammasome can shear IL-1β and IL-18 precursors into mature IL-1β and IL-18 through activated caspase-1, which in turn activates downstream signaling pathways and produces a mass of inflammatory mediators, causing severe inflammatory responses in the body and playing an important role in the pathogenesis of cardiovascular and cerebrovascular disease such as AMI and ischemic brain infarction.^[19]^ Inflammatory factors such as IL-1β and IL-6 play an essential part in the development of myocardial ischemia-reperfusion injury.^[20]^ HMGB1 has been shown to be an abundant non-histone nuclear protein, known for its fast migration rate in polyacrylamide gel electrophoresis. HMGB1 promotes gene transcription in cells, maintains nucleosomes, facilitates deoxyribonucleic acid recombination, replication and folding, and regulates transcriptional processes.^[21]^ HMGB1 plays a crucial role in many serious inflammatory diseases such as septicemia, acute lung injury and rheumatoid arthritis when it is released extracellularly and becomes a danger signal for the body.^[22–24]^ Recent studies have revealed that HMGB1 plays an equally important role in the development and progression of cardiovascular diseases such as myocardial infarction and myocardial ischemia-reperfusion injury.^[25,26]^ Recently, our study found that serum HMGB1 expression level was considerably higher in patients with stable angina and unstable angina and was positively correlated with the degree of coronary artery stenosis. Dong et al demonstrated that the expression of HMGB1 in ischemia-reperfused rat heart was positively correlated with the expression of IL-1β and IL-6, further suggesting that HMGB1 can stimulate the expression and release of multiple inflammatory factors.^[27]^ In addition, it has been demonstrated that IL-6 stimulates the release of HMGB1 from macrophages in a time and dose dependent manner, suggesting that the 2 can form a positive feedback and together promote the development of inflammation.^[28]^ GZMA is the most abundant protease in cytotoxic particles and is the main mediator of in vitro toxicity.^[29]^ GZMA has been proven to activate the non-caspase-dependent apoptosis pathway through cleavage of the mitochondrial protein NDUFS3, resulting in the generation of reactive oxygen species.^[30]^ In addition, GZMA directly stimulates the production and release of IL-6, IL-8, and TNF-α of monocytes and macrophages from human peripheral blood.^[31,32]^ TNF-α has a variety of biological activities and is one of the first inflammatory factors produced by the body after stimulation. It can cause endothelial cell damage, increase the expression of adhesion molecules and other inflammatory mediators, and play an important role in the development of Cardiovascular and cerebrovascular diseases.^[33]^ Bax, a Bcl-2 homologue, inhibits the action of Bcl-2 and promotes apoptosis, while Bcl-2 inhibits apoptosis. It was found that the ratio of Bcl-2 to Bax (Bcl-2/Bax) determines apoptosis. When Bax expression is higher, Bax/Bax homodimers are formed to promote apoptosis, and when Bcl-2 expression is higher, Bcl-2/Bcl-2 homodimers are formed to suppress apoptosis.^[34]^ Other studies have also demonstrated that the degree of apoptosis is related to the Bcl-2/Bax ratio in cardiomyocytes, with an increase in the Bcl-2/Bax ratio which inhibit apoptosis, and a decrease in the Bcl-2/Bax ratio which promote apoptosis in cardiomyocytes.^[35]^ p53, a target gene for Bax and Bcl-2, is also an important regulator of the programmed cell death (PCD), and upregulation of p53 protein expression will promote apoptosis.^[36]^ Bcl-2 and p53 proteins interact in an antagonistic manner in apoptosis, with Bcl-2 specifically inhibiting p53-mediated apoptosis of cells.^[37]^

In this study, by clustering and typing the pyroptosis-related genes, we found that type A had better pyroptosis score than type B, and most of the pyroptosis-related genes showed high expression in subtype B. Immune cell infiltration analysis revealed that cells such as eosinophils, γδ T cells, immature B cells, MDSC, macrophages, mast cells, monocytes, natural killer cells, neutrophils, plasmacytoid dendritic cells, regulatory T cells, T follicular helper cells, type 1 helper T cells differed sharply between the 2 subtypes, suggesting that the number of immune cells in AMI patients is altered by the influence of pyroptosis-related genes in vivo. For example, eosinophil deficiency leads to decreased in anti-inflammatory macrophages, increased myocardial inflammation, increased scarring and deterioration of myocardial structure and function.^[38]^ It has been reported that MDSC is involved in the host innate immune and inflammatory response through the secretion of arginase 1 (ARG1) and inducible nitric oxide synthase.^[39]^

High expression of dendritic cell-associated C-type lectin-1, CXC chemokine receptor 7, Annexin A1 and cluster of differentiation 226 on the surface of macrophages after infarction promotes the polarization of macrophages towards M1 phenotype and enhances the infiltration of neutrophils and γδ T cells after AMI.^[40,41]^ KEGG enrichment analysis revealed that the differential genes were mostly enriched in NF-kappa B signaling pathway, neutrophil extracellular trap formation, lipid and atherosclerosis, IL-17 signaling pathway, hematopoietic cell lineage, C-type lectin receptor signaling pathway, TNF signaling pathway, cytokine-cytokine receptor interaction, phagosome, NOD-like receptor signaling pathway, Toll-like receptor signaling pathway. It is notable that the formation of neutrophil extracellular traps (NETs) is an important factor in the progression of the disease. Neutrophils are known to be part of the first line of defense against invading pathogens and are an important component of nonspecific immunity, eliminating sterile inflammation through chemotaxis, phagocytosis and degranulation. After stimulation of neutrophils by pyroptosis signals, the cell membrane is disrupted, releasing cytoplasm and depolymerized chromatin.^[42]^ Therefore, the formation of neutrophil extracellular trap formation is an important pathological change after neutrophil pyroptosis. Regarding that neutrophil-derived serine proteases and nucleosomes from NETs may induce large vessel thrombosis leading to AMI and stroke, which significantly promotes thrombosis of NETs in both humans and animals.^[43]^ Th17 cells, a newly discovered CD4 + T lymphocyte subpopulation in recent years, can secrete not only IFN-γ but also IL-17, which is the main effector of Th17 cell subpopulation. It was found that IL-17 and IFN-γ produced by T lymphocytes in atherosclerotic lesions can lead to an inflammatory state of vascular smooth muscle cells, suggesting that Th17 cells and their secreted IL-17 have a pathogenic role in the development and formation of coronary heart disease.^[44]^ Toll-like Receptors (TLRs) are a family of pattern recognition receptors that regulate immune response and inflammation in vivo, recognizing a variety of pathogenic pattern molecules, influencing the differentiation and maturation of immune cells and the activation of inflammatory cells through downstream signal transduction cascades. TLR4 is the most closely related member of TLRs family to the inflammatory response, which can activate the transcription factor nuclear factor κ-light-chain-enhancer of activated B cells through MyD88 and non-MyD88 pathways, and the activated nuclear factor κ-light-chain-enhancer of activated B cells translocates into the nucleus and regulates the expression of various inflammatory mediators.^[45]^ In summary pyroptosis-related genes may influence the pathogenesis and prognosis of AMI patients by interfering with the above immune cells and signaling pathways.

In conclusion, we identified 19 genes characteristic of pyroptosis in AMI patients by bioinformatics and analyzed their biological roles of AMI.

The results of our nomogram are of great value for clinical prediction of AMI, and the 19 pyroptosis-related signature genes may provide directions for clinical targeted drug development.

## 5. Conclusion

In this study, we used RF and SVM algorithms to determine 19 pyroptosis-related genes that strongly contribute to inflammatory injury after AMI in patients. We also constructed a series of by nomogram related to pyroptosis-related genes to predict the risk of developing AMI.

## Acknowledgments

We thank the authors who provided the GEO public datasets.

## Author contributions

**Data curation:** Jianlong Xiong, Yan Zhang, Liubo Xiang.

**Formal analysis:** Xiaoman Xiong, Xueying CUI.

**Project administration:** TAO Xu.

**Software:** Jianlong Xiong, Yan Zhang, Liubo Xiang.

**Writing – original draft:** Wu Huan, Xiaoman Xiong, Xueying CUI.

**Writing – review & editing:** TAO Xu.
